# Penile Fracture Secondary to Gunshot Injury: A Multidisciplinary Approach and Exceptional Surgical Management in an Uncommon Case

**DOI:** 10.7759/cureus.52928

**Published:** 2024-01-25

**Authors:** Javier Meza-Hernandez, Christian Sánchez-Treviño, Marco Antonio Ascencio-Martínez, Alejandra Nuñez-Venzor

**Affiliations:** 1 Department of General and Endoscopic Surgery, Hospital General Dr. Manuel Gea González, Mexico City, MEX; 2 Department of Urology, Hospital General Dr. Manuel Gea González, Mexico City, MEX

**Keywords:** uncommon case, surgical management, multidisciplinary approach, gunshot wound, penile fracture

## Abstract

Penile fractures happen when the tunica albuginea is forcefully torn during intense sexual activity or vigorous masturbation. Gunshot-induced cases are extremely rare. Diagnosis, often requiring surgical exploration, poses challenges due to the condition's rarity and severity. We report a complex case of a patient with multiple gunshot wounds, highlighting the need for a multidisciplinary approach for abdominal and genitourinary regions.

A 24-year-old male presented to the emergency department with multiple gunshot wounds to the anterior thoracic and abdominal walls, inguinal region, penis, and lower extremities. Despite multiple gunshot wounds, the patient maintained hemodynamic stability during physical examination. No imaging study was performed since surgical management was decided due to the presence of hematemesis. During exploratory laparotomy, a 2 cm stomach lesion was found and repaired by the general surgery team. Urology then addressed genital trauma, identifying and fixing a 1 cm tunica albuginea defect in each corpora cavernosa, achieving bilateral penile fracture repair. The patient was discharged after eight days of hospitalization, with adequate oral intake and urinating. Fifty-two days later, he persists with mild erectile dysfunction (International Index of Erectile Function-5 score: 17 points).

This unique case involving a gunshot-induced penile fracture alongside abdominal and several other injuries was successfully managed through a multidisciplinary approach. As these lesions are rare, prompt treatment with standardized surgical procedures for civilian cases is crucial for optimal outcomes.

## Introduction

Penile fracture is a rare occurrence in trauma cases, with a frequency of one in 175,000. It is defined as the traumatic tearing of the tunica albuginea in the corpora cavernosum. Common triggers include instances like the penis hitting the perineum during intercourse or vigorous masturbation [1]. Extremely rare cases include those caused by gunshot wounds (GSWs), with only a few cases reported in the literature from 1963 to 2023 [2-6].

Penile fractures usually exhibit consistent clinical signs, such as a hematoma localized to the penis, identified in approximately 90% of cases during physical examination [7]. Diagnosis involves clinical assessment with additional use of imaging modalities to confirm the diagnosis, such as ultrasound, cavernosography, retrograde urethrography, and magnetic resonance imaging (MRI) [1].

The predominant treatment approach is immediate surgical exploration, performed in 95.4% of patients. Alternatively, in 4.6% of cases, conservative management may be contemplated, involving local ice application, compressive bandaging, anti-inflammatories, and antibiotics [1]. It is worth noting that the rarity of penile fractures can pose a challenge in providing prompt and conclusive care, as experience in managing such cases may be limited, and a multidisciplinary approach is required due to the severity in which the patient presents [8].

We present a complex case involving a patient with multiple gunshot wounds in both the abdomen and genital region, necessitating a coordinated, multidisciplinary approach by the general surgery and urology departments.

## Case presentation

A 24-year-old male presented to the emergency department with no significant medical history, experiencing multiple GSWs to the anterior thoracic and abdominal walls, inguinal region, penis, and lower extremities. A trauma code was activated.

**Figure 1:**
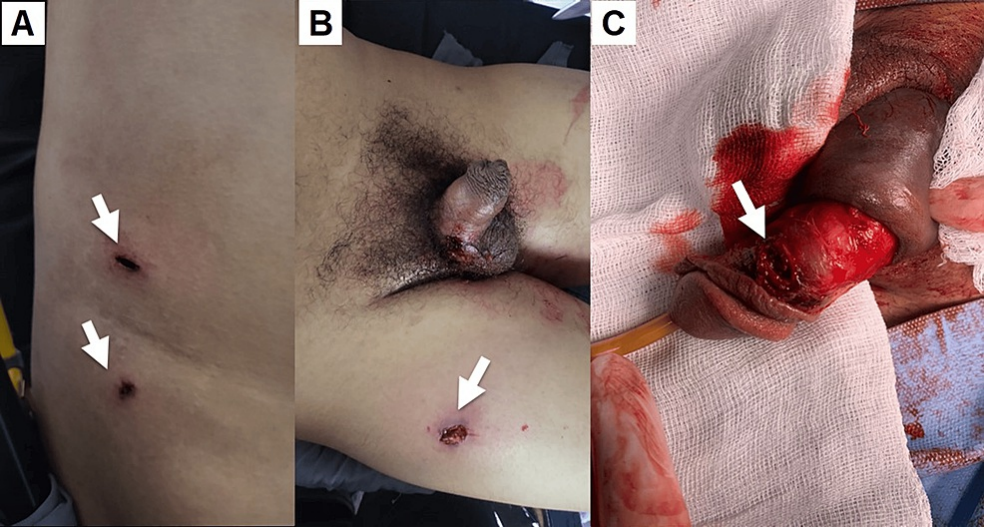
Abdominal and inguinal lesions. (A) Right flank abdominal GSW (arrows). (B) Right thigh entry GSW (arrow). (C) Penile left entry GSW (arrow). GSW: gunshot wounds.

During physical examination, the patient was hemodynamically stable despite sustaining multiple GSWs. He did not present urethrorrhagia, acute urinary retention, or classic signs of penile fracture (such as the “eggplant deformity”), but presented with hematemesis. Physical findings revealed a 2 cm penetrating wound in the left hemithorax; two abdominal 2 cm lesions on the right flank, each with projectile penetration; one GSW at the inguinal level, in the left fossa; in the lower extremities, a 1 cm GSW was found in the right thigh in the lateral aspect, with an exit orifice on the medial part of the thigh. The penis displayed a 0.5 cm lesion on the lateral edge with bleeding within the genitourinary region (Figure 1).

Additionally, a GSW was detected in the posterior region of the left ankle. Laboratory results revealed respiratory acidosis, leukocytosis with a predominant neutrophilic response, and elevated C-reactive protein, consistent with anticipated outcomes in the aftermath of trauma, including anemia. No imaging modality was performed.

The patient underwent an exploratory laparotomy led by the general surgery team, due to penetrating wounds in the abdominal cavity and hematemesis finding. Before surgery, an 18 French Foley transurethral catheter was placed without difficulty. After its placement, there was unobstructed urinary return without urethrorrhagia or hematuria. A midline supra and infraumbilical incision revealed approximately 1500 cc of hemoperitoneum. A 2 cm grade II stomach lesion on both the posterior and anterior aspects of the stomach body was discovered and repaired with vertical Sarnoff stitches using 3-0 Vicryl. Additionally, a lesion in the left hypochondrium wall, coupled with a 19th rib costal fistula, was repaired using continuous sutures of 3-0 Vicryl. Evisceration and exploration of the small intestine were conducted, followed by a thorough examination of the colon, revealing no evidence of lesions. A left endopleural catheter was also placed during surgery due to a thoracic penetrating injury, ruling out a hemopneumothorax.

**Figure 2:**
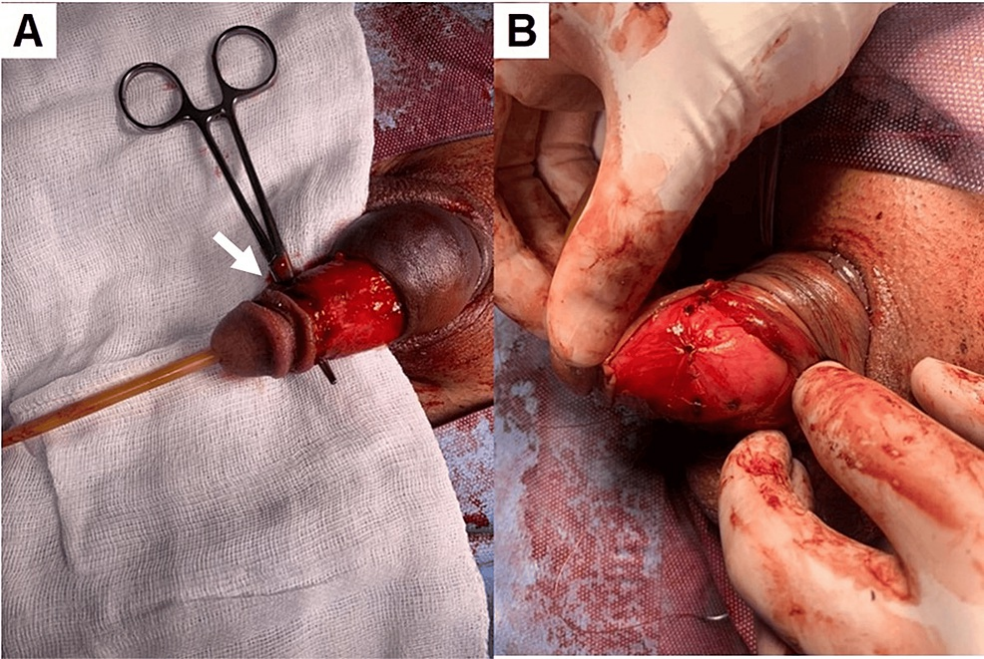
Surgical exploration of the penis. (A) Bilateral fracture of the corpora cavernosa demonstrating the bullet’s path where entry wound (arrow) and exit wound are observed. (B) Bilateral penile fracture repair.

Urology was consulted intraoperatively to evaluate genitourinary trauma. First, a retroperitoneum exploration during exploratory laparotomy with the general surgery team was conducted, ruling out renal compromise. Then the genital trauma was addressed; surgery involved a surgical exploration of the penis. After exposure of both corpus cavernosum and corpus spongiosum, a bilateral 1 cm solution of continuity in the tunica albuginea just below the glans was identified, leading to bilateral penile fracture repair with a 3-0 Monocryl two planes stitch, ensuring hemostasis. The urethra was intact. For closure and stitching the skin around the penis to the glans, 3-0 Chromic sutures were used (Figure 2).

**Figure 3:**
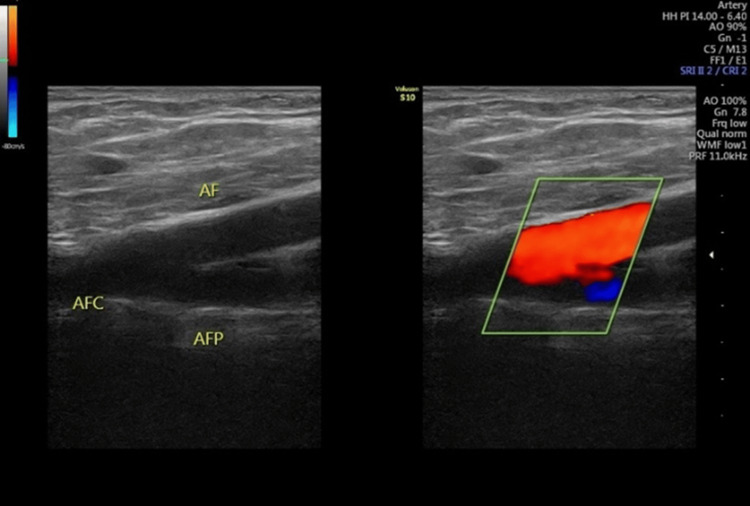
Doppler ultrasound of the common femoral artery, femoral artery, deep femoral artery, popliteal artery, anterior tibial artery, and posterior tibial artery with negative findings of arterial thrombosis. AFC: common femoral artery; AF: femoral artery; AFP: deep femoral artery.

Topical mupirocin and a compressive dressing were applied. Cutaneous wounds on both sides of the base of the penis were sutured with 3-0 nylon, covered with a sterile dressing, ending the procedure. Although the patient did not experience hemodynamic instability, the procedure was still performed this way to do the penis reparation with more precision. After surgery, a Doppler ultrasound of the left pelvic limb arteries was performed without findings for arterial thrombosis (Figure 3).

The patient was discharged eight days after surgery, with successful removal of transurethral, and was referred to primary care for follow-up. Fifty-two days after trauma, he scored 17 points on the International Index of Erectile Function (IIEF-5), interpreted as mild erectile dysfunction (17-21) [9].

## Discussion

Penile fractures are urological emergencies demanding immediate surgical exploration for the preservation and restoration of form and function. Urgent intervention is crucial to minimize bleeding, repair damaged structures, and address potential long-term complications. Of notable concern is addressing associated urethral damage, observed in 11% to 29% of penetrating penile injuries. While historical management leaned toward conservative approaches, the significant morbidity associated with this approach (up to 30%), including erectile dysfunction (ED), Peyronie's disease with palpable fibrous plaques, painful erections, curvature, and infected hematomas, underscores the necessity for a more proactive surgical strategy [6,10].

In our case, we performed successful management in a challenging surgery involving both abdominal and genital trauma. Besides the stomach reparation of the abdominal cavity, we left in our patient an endopleural probe due to the evidence of the exit of the projectile at the level of the 10th intercostal space in the left anterior axillary line, giving priority to the patient's life and the organic function of the digestive tract. Subsequently, the penile fracture was surgically approached at the same surgical time. It is important to note that the integrity of the urethra was verified so that the raffia of the corpora cavernosa could be performed. An important point to discuss, highlighting the rarity of the case, is that in the vast majority of instances, a penile fracture occurs when the penis is erect, which was not the case in this situation.

Given the presence of a penetrating injury through Buck's fascia accompanied by an expanding hematoma, prompt surgical intervention by the urology service is recommended, bypassing the wait for imaging results. The surgical procedure involves the removal of the hematoma, achieving hemostasis, and a meticulous assessment of the penile corpora by injecting saline into the cavernosa. Any observed damage or leakage can be addressed through the application of absorbable sutures discreetly placed beneath Buck's fascia [1]. Reports from various series suggest a favorable prognosis with this approach, often yielding positive outcomes and a substantial likelihood of regaining erectile function within six weeks postoperatively [10].

In cases of mild ED, the initial approach involves lifestyle modifications and the use of phosphodiesterase type 5 (PDE5) inhibitors like sildenafil. Less invasive options like vacuum erection devices (VED) can be considered. In persistent cases, exploration of intraurethral suppositories (IUS) is recommended, reserving surgery or penile implants for severe situations [8]. Even though we could have continued the patient's treatment, he decided to transfer to another institution due to his insurance affiliation with that organization for follow-up of ED.

## Conclusions

This case highlights a rare occurrence of a penile fracture resulting from GSWs, accompanied by multiple abdominal and genital injuries. The successful management of this complex scenario was achieved through a crucial multidisciplinary approach. Given the rarity of these cases in the civilian environment, it is important to standardize surgical management to treat them as quickly and effectively as possible.
